# Epilepsy–heart syndrome: Concept, clinical context, and opportunity for integrated care

**DOI:** 10.1111/epi.18356

**Published:** 2025-03-13

**Authors:** Gashirai K. Mbizvo, Iain E. Buchan, Anthony G. Marson, Gregory Y. H. Lip

**Affiliations:** ^1^ Liverpool Centre for Cardiovascular Science at University of Liverpool Liverpool John Moores University and Liverpool Heart and Chest Hospital Liverpool UK; ^2^ Pharmacology and Therapeutics, Institute of Systems, Molecular, and Integrative Biology University of Liverpool Liverpool UK; ^3^ Walton Centre National Health Service (NHS) Foundation Trust Liverpool UK; ^4^ Department of Public Health, Policy, and Systems, Institute of Population Health University of Liverpool Liverpool UK; ^5^ Danish Center for Health Services Research, Department of Clinical Medicine Aalborg University Aalborg Denmark

**Keywords:** Cardiovascular disease, Epileptic heart, Ictal asystole, Seizures, SUDEP

## Abstract

In this concept paper, we introduce epilepsy‐heart syndrome as a shared burden of illness between epilepsy and cardiac disorders. This pragmatic definition is agnostic of which condition came first (the epilepsy or the cardiac disorder), recognising that these conditions can each serve as a risk factor for the other owing to a bidirectional relationship that exists between the brain and the heart. To provide clinical context, we include ictal asystole as an example phenotype of epilepsy‐heart syndrome. We highlight evidence of patients with ictal asystole coming to harm owing to the failure of integrated care between neurology and cardiology. This underscores epilepsy‐heart syndrome as an unmet need for collaborative care between neurology and cardiology. To address this, we propose a framework for integrated care, drawing upon our own centre's recently established and successful multidisciplinary team meeting (MDT) between neurologists and cardiologists, our joint cardiology‐neurology PhD programme, and our work developing a joint national guideline on ictal asystole management between the Association of British Neurologists (ABN) and the British Heart Rhythm Society (BHRS).

**Key points**

- Epilepsy–heart syndrome is a shared burden of illness between epilepsy and cardiac disorders, agnostic of the chronicity of epilepsy.
- Ictal asystole is one example of an epilepsy–heart syndrome phenotype.
- The failure of integrated care between neurology and cardiology has led to patients with ictal asystole coming to harm.
- We encourage colleagues to consider integrating neurology and cardiology services to optimize epilepsy–heart syndrome care.
- Our epilepsy–heart syndrome multidisciplinary team meeting congregates neurologists and cardiologists and is one example of an initiative to consider.

## 1 CONCEPT

The epileptic heart concept proposed by Verrier and colleagues in 2020 describes “heart and coronary vasculature damaged by chronic epilepsy as a result of repeated surges in catecholamines and hypoxemia leading to electrical and mechanical dysfunction.”^1^, ^2^ Herein, we propose a broader epilepsy–heart syndrome,^3^ not only encompassing the epileptic heart but also recognizing the syndromic burden of illness interconnecting seizures and high levels of cardiovascular diseases experienced by people with epilepsy (PWE). This definition goes beyond a unidirectional mechanism of cardiac damage from chronic epilepsy. Instead, it reflects growing evidence of a bidirectional relationship between epilepsy and cardiac diseases.^4^, ^5^, ^6^ PWE suffer a sixfold increased risk of sudden cardiac death or cardiac arrest compared to people without epilepsy,^7^, ^8^ fivefold increased risk of myocardial infarction,^9^ fourfold increased risk of ischemic heart disease,^10^ two‐ to threefold increased risk of all‐cause deaths,^3^, ^7^, ^11^ and nearly twofold increased risk of ventricular arrhythmias,^12^ atrial fibrillation (AF),^12^ and heart failure.^13^, ^14^ However, people with cardiac diseases including AF, flutter, valvular heart disease, hypertension, and hyperlipidemia have an increased risk of developing epilepsy,^5^, ^6^ and this can be independent of stroke.^4^ Furthermore, PWE with ictal asystole, for example, do not necessarily have heart or coronary vasculature damage from chronic epilepsy.^15^, ^16^ Instead, individual seizures trigger asystole through activation of the central autonomic network,^17^ making it a cardiac complication that can, and often does, occur early in epilepsy.^15^, ^16^ This means ictal asystole does not require chronic epilepsy, and the heart and coronary vasculature may otherwise be normal. Whereas Verrier and colleagues have helpfully defined an epileptic heart syndrome that is reliant on chronic epilepsy and resultant cardiac damage,^18^ we define epilepsy–heart syndrome as a shared burden of illness between epilepsy and cardiac disorders agnostic of the direction of effect or chronicity of epilepsy (Table 1). This definition includes both PWE who develop cardiac disorders early and people with cardiac disorders who develop epilepsy. Our broader definition is more inclusive and would capture, for example, PWE with ictal asystole in early epilepsy with a normal heart and coronary vasculature. This is crucial, as ictal asystole is often poorly recognized and managed owing to failures of integrated care between neurology and cardiology,^15^, ^16^ as we highlight later. Our definition is designed to be pragmatic for clinicians and patients, to unlock greater potential for integrated care between neurology and cardiology. It ensures those with incident or early epilepsy and cardiac complications (such as ictal asystole) and/or those with preexisting cardiovascular disorders (such as AF, flutter, hypertension, or hyperlipidemia) who develop epilepsy are recognized by both neurology and cardiology and given equal priority to those with a traditional epileptic heart by the Verrier definition. This syndromic approach anticipates the growing complexity and understanding of an epilepsy and cardiac disorders phenotype.

**TABLE 1:** Diagnostic criteria for epilepsy–heart syndrome.

Epilepsy–heart syndrome is defined as the coexistence of epilepsy and a cardiac disorder, irrespective of the direction of effect or chronicity of epilepsy. A diagnosis of epilepsy–heart syndrome requires BOTH of the following criteria (1 and 2) occurring in any order:	
Criterion	Diagnostic requirement
Epilepsy With or without suspected antiseizure medication side effects on cardiovascular health^a^	*Must meet latest ILAE diagnostic criteria for epilepsy (2017), currently defined as*: At least two unprovoked seizures (or reflex seizures) occurring >24 h apart, OR One unprovoked (or reflex) seizure with a high recurrence risk (≥60%), based on clinical, EEG, and imaging features, OR Diagnosis of an epilepsy syndrome (e.g., juvenile myoclonic epilepsy, Dravet syndrome, or Lennox–Gastaut syndrome)
2 Presence of a recognized cardiac disorder With or without suspected cardiac medication proconvulsant side effects^b^	*Must meet latest ESC/AHA/ACC or other established diagnostic criteria for a recognized cardiac disorder such as*: Structural heart disease Heart failure (e.g., HFrEF or HFpEF, with LVEF ≤ 40% for HFrEF) Ischemic heart disease (e.g., confirmed via stress testing, coronary angiography, or imaging) Myocardial infarction (including ST‐segment depression, elevation, or Q‐waves) Valvular heart disease (e.g., meeting severity thresholds from ESC/AHA guidelines) Diastolic dysfunction on echocardiography (e.g., increased left ventricle stiffness, elevated left ventricular diastolic filling pressure, or increased left atrial volume) Arrhythmias Cardiac arrest Atrial fibrillation/flutter (e.g., confirmed via ECG) Ventricular arrhythmias (e.g., ventricular tachycardia, fibrillation, Brugada syndrome) LQTS (e.g., QTc >480 ms or LQTS diagnostic score >3 per ESC 2022 guidelines) Cardiac conduction abnormalities (e.g., prolonged PR interval, widened QRS) Cardiac autonomic dysfunction Ictal asystole or postictal asystole (e.g., confirmed via ECG, loop recorder, or Holter monitor) Altered heart rate variability (e.g., rMSSD < 27 ± 12 ms, LF/HF ratio > 1.5–2.0) T‐wave alternans ≥ 47 μV (marker of cardiac electrical instability, linked to seizure activity) Hyperlipidemia, atherosclerosis, or hypertension Triglycerides (e.g., >149 mg/dL; HDL < 40 mg/dL [men], HDL < 50 mg/dL [women]) Consider ultrasound measurement of CIMT to assess vascular risk Blood pressure (e.g., sustained ≥140/90 mmHg)

Abbreviations: ACC, American College of Cardiology; AHA, American Heart Association; ASM, antiseizure medication; CIMT, carotid intima–media thickness; ECG, electrocardiogram; EEG, electroencephalogram; ESC, European Society of Cardiology; HF, high frequency; HFpEF, heart failure with preserved ejection fraction; HFrEF, heart failure with reduced ejection fraction; ILAE, International League Against Epilepsy; LF, low frequency; LQTS, long QT syndrome; LVEF, left ventricular ejection fraction; QTc, corrected QT interval; rMSSD, root mean square of the successive differences.

^a^ Enzyme‐Inducing ASMs (e.g., carbamazepine, phenytoin, phenobarbital) → accelerated atherosclerosis and increased lipid levels, raising ischemic heart disease risk; sodium channel‐blocking ASMs (e.g., carbamazepine, phenytoin, lamotrigine) → avoid in Brugada syndrome; valproate → obesity, insulin resistance, metabolic syndrome, increasing hypertension and cardiovascular risk; Cannabidiol for epilepsy → avoid in Brugada syndrome.

^b^ Diuretics (e.g., furosemide, thiazides, spironolactone) → hyponatremia, lowering seizure threshold; calcium channel blockers (e.g., intravenous verapamil) → theoretical risk of seizure provocation; antiarrhythmics (e.g., flecainide) → sodium channel blockade with proconvulsant effects.

Mechanisms underlying the complex interplay between epilepsy and cardiac diseases include repeated seizure‐induced myocardial hypoxia and ischemia,^19^, ^20^ cardiotoxic effects of excess catecholamines during seizures,^17^, ^21^, ^22^ and adverse antiseizure medication (ASM) effects on the cardiovascular system through weight gain,^23^ enzyme induction,^24^ hyperlipidemia,^25^, ^26^ and proarrhythmogenic sodium channel blockade.^27^ Autonomic dysfunction during or after seizures may cause cardiac and pulmonary changes that contribute to sudden unexplained death in epilepsy.^28^, ^29^ In the other direction, cardiac diseases such as AF, hypertension, and hyperlipidemia can impair cerebral perfusion, altering neuronal excitability and promoting neurodegeneration.^30^, ^31^, ^32^ The renin–angiotensin system in the brain is implicated in diverse neurological functions and is emerging as a theoretical mechanism potentially linking hypertension to epilepsy.^6^ Furthermore, cardiac diseases are associated with chronic low‐grade inflammation through, for example, elevated cytokines like IL‐6 and TNF‐α,^33^, ^34^ which disrupts the blood–brain barrier and has been implicated in the development of epilepsy.^35^ Treatments for cardiac diseases, such as diuretics, may also increase susceptibility to seizures through electrolyte imbalances.^36^

Cardiac imaging and postmortem assessment of cardiac tissue in PWE reveals changes in myocardial structure, including myocyte vacuolization,^37^ interstitial fibrosis,^37^, ^38^ contraction‐band necrosis,^18^ increased myocardial stiffness,^39^ and systolic or diastolic dysfunction.^40^ In the other direction, cardiac dysfunction contributes independently to the development of cerebral magnetic resonance imaging (MRI) abnormalities in, for example, patients with heart failure.^41^, ^42^ Interictal electrocardiographic (ECG) abnormalities found in PWE include first‐degree atrioventricular block and poor R‐wave progression,^43^ both markers of cardiac disease. Postictal ECG changes may reveal T‐wave alternans, an independent marker of risk of sudden cardiac death or instability.^44^, ^45^ In the other direction, electroencephalographic (EEG) abnormalities in heart failure are similar to those observed in cognitively impaired patients, suggesting an underlying high brain sensitivity to cardiac disease that could have implications for seizure risk.^32^, ^46^ Atherosclerotic disease is increased in PWE,^47^ as is obesity,^48^ hypertension,^49^ and diabetes,^49^ all of which worsen cardiovascular health.^5^ Furthermore, sodium and potassium ion channels regulate the functioning of both neurons and cardiac cells. Mutations in ion channel genes such as *SCN5A*, *KCNA1*, and *KCNH2* can manifest in both epilepsy and cardiac arrhythmia, illustrating the potential for a shared genetic basis for epilepsy and cardiac diseases.^5^, ^50^

## 2 CLINICAL CONTEXT

Ictal asystole is one example of an epilepsy–heart syndrome phenotype in which patients are coming to harm owing to lack of integrated care between neurology and cardiology.^15^, ^16^ In 2019, we reported two cases of adults presenting with transient loss of consciousness (TLoC) followed by a rapid recovery.^16^ Careful history‐taking revealed a stereotyped prodrome of déjà vu, raising the possibility of these events representing syncope as symptomatic of a focal seizure, rather than being simple syncope. The patients were commenced on ASMs at the same time as having cardiac monitoring organized. This confirmed asystole during the seizure symptoms, resulting in TLoC. It was assumed that the cardiac arrhythmia explained the entire picture, a permanent pacemaker (PPM) was inserted, and the ASMs were withdrawn in one patient (who was also told they could now drive) and not commenced in the other. However, both patients subsequently presented with seizures, including generalized tonic–clonic seizures, despite a functioning pacemaker. The penny then dropped, so to speak, that this was ictal asystole. The latter is a condition in which seizures (usually originating in the temporal lobe) cause asystole through stimulating the central autonomic network and evoking a vasovagal reflex via catecholamine release.^17^ The patients were commenced on ASMs and subsequently remained seizure‐free.

**FIGURE 1:**
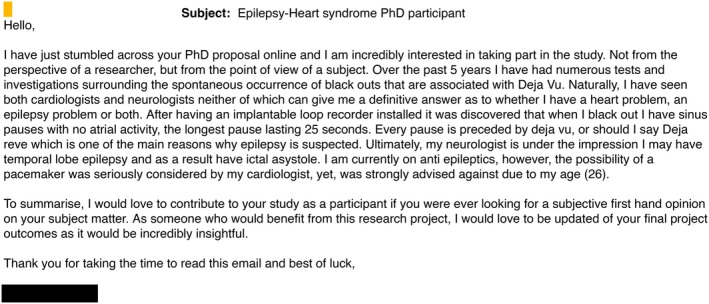
Patient perspective correspondence received on the subject of epilepsy–heart syndrome from a member of the public (included with permission).

We have since been contacted by several concerned members of the public with similar experiences of disconnected care between neurology and cardiology over ictal asystole, and we have published their patient perspectives as evidence of this (see letter in the appendix of Mbizvo et al.,^16^ the letter in Mbizvo et al.,^15^ and a more recent patient perspective received in Figure 1 [included with permission]). Part of the conundrum with ictal asystole management is that pacemakers are not without complication, including ongoing infection risks and a need to surgically replace the device when its battery runs flat. Therefore, in our experience, cardiologists do not normally wish to insert pacemakers in everyone with ictal asystole, instead proposing they should be treated with ASMs alone. They are particularly concerned about inserting pacemakers in young adults with ictal asystole, as they would require the device to stay in situ for many years, with frequent potentially risky replacements during that period. Neurologists, however, raise concern that the ASMs do not necessarily stop all seizures,^51^ meaning patients remain at risk of breakthrough seizure‐related asystole, injury, and death. The unanswered questions for ictal asystole care remain as follows:

- Is it acceptable to manage ictal asystole with ASMs alone?
- Must you use ASMs and a pacemaker?
- If using both ASMs and a pacemaker, would you apply treatment simultaneously or sequentially? Following what criteria?

These are questions that plague both neurologists and cardiologists, who have differing opinions on them.^15^, ^16^ As a result, there remains substantial heterogeneity in clinical practice on this nationally and internationally, and patients are coming to harm as a result.^15^, ^16^ Work is needed to determine the level of risk associated with each of these three policies (ASMs alone vs. ASMs combined with an early or late pacemaker) and to gather feedback from both physicians and patients on whether these risks are acceptable to them. Discrete choice experiments may support this process, as they can highlight differences in how physicians and patients prioritize acceptable risk for the same decision.^52^ This is vital to ensure physicians do not deny treatment (such as a pacemaker) based on a mistaken assumption that their own assessment of acceptable risk aligns with that of their patient.

## 3 OPPORTUNITIES

There is an opportunity to integrate neurology and cardiology services to optimize care for patients with epilepsy–heart syndrome. At our center, we recently commenced an epilepsy–heart syndrome multidisciplinary team (MDT) meeting. This brings together neurologists, cardiologists, specialist nurses, electrophysiologists, and trainees in these specialties once a month. It has opened up a direct channel of communication between neurology and cardiology, allowing rapid review of ECGs and opinions around diagnostic uncertainty on whether cases represent a cardiac disorder, epilepsy, or both. Cardiologists have rapid access to a specialist epilepsy opinion and tests such as epilepsy protocol brain MRI and EEG. Similarly, neurologists have rapid access to specialist cardiology opinions, complex ECG reviews, and tests such as tilt table, Holter monitors, and loop recorders. The cases discussed have included PWE with ictal asystole, AF, ventricular arrhythmias, intraventricular conduction delays, Brugada syndrome, prolonged QT, and unexplained nocturnal sinus tachycardias. This has facilitated early application of multiday patch‐based ECGs and/or loop recorder insertion, PPMs, genetic testing for inherited cardiac disorders, advice on ASM use (particularly which to avoid), and ongoing shared care of patients. We now have consensus‐level MDT agreement between neurology and cardiology that leadless pacemakers will be considered for young adults with ictal asystole at our center. Leadless pacing is a recent technological advance that potentially avoids the 5%–6% annual risk of long‐term complications associated with pacing leads inserted with traditional pacemakers.^53^, ^54^ In particular, the prevalence of leadless device infections is low, as the principal sources of infection—the subdermal surgical pocket and pacemaker leads—are absent.^53^ Overall, patients discussed at our epilepsy–heart syndrome MDT have reported subjective improvement in their experience and journey between neurology and cardiology. We will use this information to leverage support and a business case for an epilepsy–heart syndrome clinic in the future, a joint affair hosted by both neurology and cardiology.

We have recently gained support from the Association of British Neurologists (ABN) to develop a joint national guideline (codeveloped by neurology and cardiology) on ictal asystole management, specifically to tackle the issues raised around ASMs versus PPMs. This will set up an infrastructure for subsequent joint guideline development on other aspects of epilepsy–heart syndrome. This will be supported by evidence generated from a joint neurology and cardiology PhD program we are hosting at our university, specifically to investigate epilepsy–heart syndrome. One of the PhD projects will seek to strengthen the evidence base and guidance around ictal asystole care by completing the following work packages:

1. Systematically review all of the literature on ictal asystole, which generally consists of case reports, case series, and narrative reviews;
2. Use this to inform and undertake a prospective observational study in collaboration with a national network of neurologists and cardiologists (including using the ABN's rare disease reporting platform [RaDAR, www.theabn.org/page/radar]), seeking information on cases of ictal asystole that members have come across and asking them how they were managed (ASMS alone? ASMs and pacemaker early or late?) and what the outcomes were (Did the patients do well? What were the issues raised?);
3. Use evidence generated from work packages 1–2 on risk to inform a series of discrete choice experiments with physicians and PWE to understand the level of acceptable and unacceptable risk regarding these three management strategies; and
4. Use evidence generated from these three work packages to underpin the development of a national guideline with the ABN and British Heart Rhythm Society (BHRS) on how ictal asystole should be managed, based on published literature and experience from colleagues around the country, taking patient and physician preferences into account.

## 4 CONCLUSIONS

**FIGURE 2:**
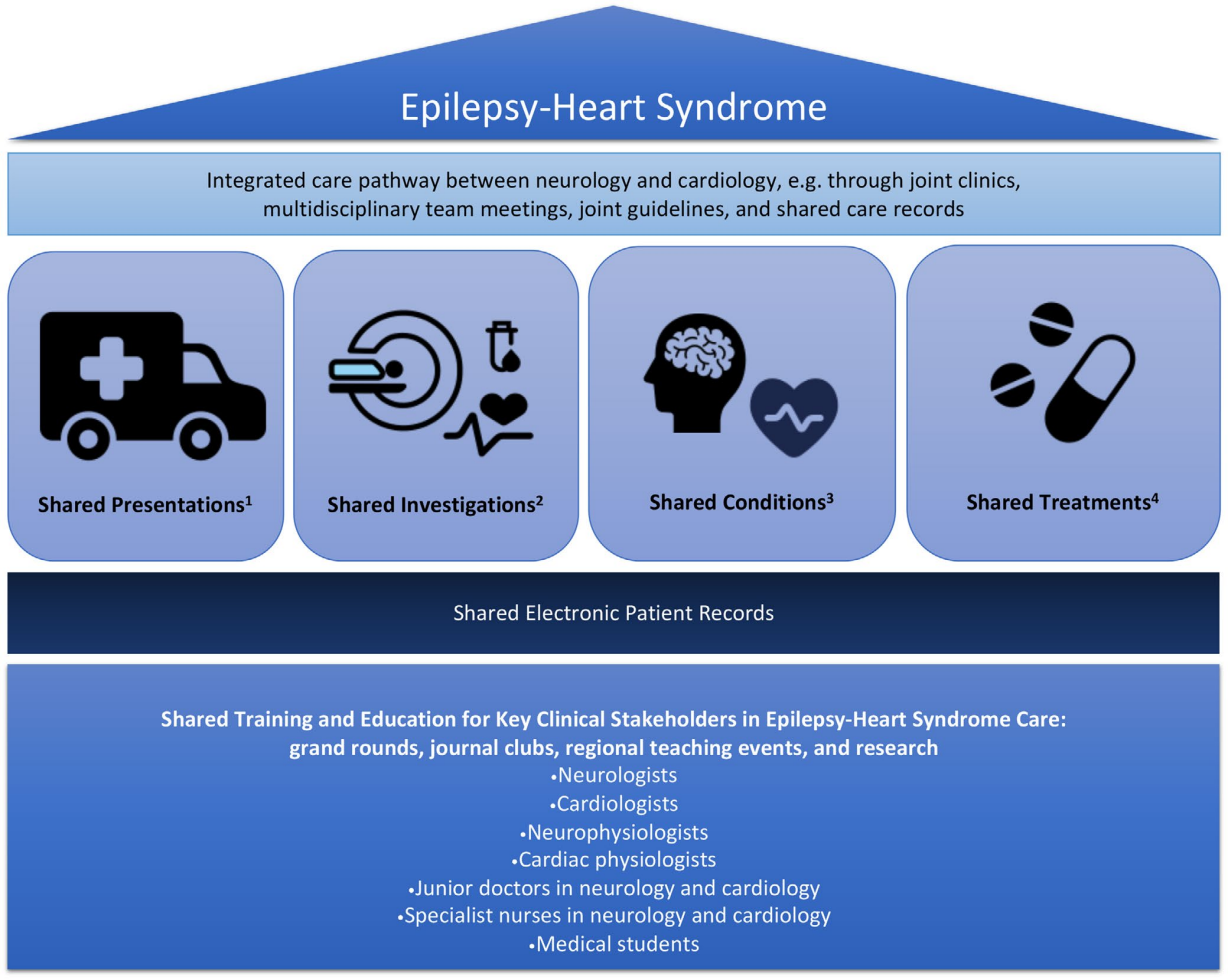
Schematic representation of shared concepts and integrated care in epilepsy–heart syndrome. ^1^Syncope, seizure, falls, dizziness or light‐headedness, exercise intolerance, palpitations, sweating or diaphoretic episodes, hypotensive episodes, postural orthostatic tachycardia, apneic episodes, sleep disturbance, headache, functional symptoms, sudden death. ^2^Electrocardiogram, electroencephalogram, video telemetry, Holter monitor, multiday patch‐based electrocardiogram, loop recorder, sleep studies, brain magnetic resonance imaging (MRI), echocardiography, cardiac computed tomography/MRI, tilt table testing, ambulatory blood pressure, serological tests (full blood count, thyroid function tests, metanephrines, genetics). ^3^Epilepsy complicated by the following: ictal asystole, postictal asystole, T‐wave alternans, atrial arrhythmias, ventricular arrhythmias, prolonged QT, cardiac conduction abnormalities (including prolonged QRS), Brugada syndrome, myocardial ischemia or infarction (including ST‐segment depression, elevation, or Q‐waves), cardiac arrest, valvular heart disease, heart failure, diastolic dysfunction (including increased left ventricle stiffness, elevated left ventricular diastolic filling pressure, or increased left atrial volume), altered autonomic tone (heart rate variability), hyperlipidemia, hypertension, obesity, young stroke, antiseizure medication adverse effects, cardiac drug adverse effects (including diuretic‐induced electrolyte abnormalities). ^4^Antiseizure medications, vagus nerve stimulation, epilepsy surgery, antiarrhythmics, pacemaker, implantable cardioverter–defibrillator, antihypertensives, antihypotensives, lipid lowering, anticoagulants, antiplatelets, diuretics.

We view epilepsy–heart syndrome as a real phenomenon that represents unmet needs for shared care between neurology and cardiology. We encourage other centers to integrate local neurology and cardiology pathways. We highlight possible approaches to consider in Figure 2. We invite further research to help fully elucidate the mechanisms underlying the bidirectional relationship between epilepsy and cardiac disorders.

## FUNDING INFORMATION

G.K.M. is supported by an NIHR Academic Clinical Lectureship (CL‐2022‐07‐002), Academy of Medical Sciences (AMS) Starter Grant for Clinical Lecturers (SGL030\1029), and Epilepsy Research Institute UK Emerging Leader Fellowship (F2401). I.E.B. is supported by an NIHR senior investigator award (NIHR205131). A.G.M. is supported by the NIHR Applied Research Collaboration North West Coast. G.Y.H.L. is an NIHR Senior Investigator and co‐principal investigator of the AFFIRMO project on multimorbidity in AF, which has received funding from the European Union's Horizon 2020 research and innovation program under grant agreement No. 899871. The views expressed in this publication are those of the authors and not necessarily those of the NIHR, the AMS, the Epilepsy Research Institute UK, Horizon Europe, or the Department of Health and Social Care. For the purpose of open access, the authors have applied a Creative Commons Attribution (CC BY) license to any Author Accepted Manuscript version arising.

## CONFLICT OF INTEREST STATEMENT

The authors report no competing interests. We confirm that we have read the Journal's position on issues involved in ethical publication and affirm that this report is consistent with those guidelines.

## Data Availability

Data sharing is not applicable to this article as no new data were created or analyzed in this study.

## References

[1] Verrier RL , Pang TD , Nearing BD , Schachter SC . Epileptic heart: a clinical syndromic approach. Epilepsia. 2021;62(8):1780–1789. 10.1111/epi.16966 34236079

[2] Verrier RL , Pang TD , Nearing BD , Schachter SC . The epileptic heart: concept and clinical evidence. Epilepsy Behav. 2020;105:106946. 32109857 10.1016/j.yebeh.2020.106946

[3] Bucci T , Mbizvo GK , Rivera‐Caravaca JM , Mayer J , Marson AG , Abdul‐Rahim AH , et al. Epilepsy‐heart syndrome: incidence and clinical outcomes of cardiac complications in patients with epilepsy. Curr Probl Cardiol. 2023;48:101868. 37295636 10.1016/j.cpcardiol.2023.101868

[4] Doege C , Luedde M , Kostev K . Atrial fibrillation is associated with a subsequent epilepsy diagnosis independent of stroke: a retrospective matched administrative cohort study on 149,632 patients. Epilepsy Behav. 2022;132:108721. 35576778 10.1016/j.yebeh.2022.108721

[5] Liu R , Tian Y , Zhang X , Zhang X , Lin Y . Bidirectional association between abnormal cardiac conditions and epilepsy: a two‐sample Mendelian randomization study. Epilepsy Behav. 2024;161:110111. 39488097 10.1016/j.yebeh.2024.110111

[6] Sun Z , Jiang T , Zhang M , Li Y , Zhang J , Sun Y , et al. Causal relationship between hypertension and epilepsy: a mendelian randomization study. Acta Epileptol. 2024;6(1):8. 40217372 10.1186/s42494-024-00152-9 PMC11960257

[7] Shah RA , Chahal CAA , Ranjha S , Sharaf Dabbagh G , Asatryan B , Limongelli I , et al. Cardiovascular disease burden, mortality, and sudden death risk in epilepsy: a UK biobank study. Can J Cardiol. 2024;40(4):688–695. 38013064 10.1016/j.cjca.2023.11.021

[8] Bardai A , Blom MT , van Noord C , Verhamme KM , Sturkenboom MC , Tan HL . Sudden cardiac death is associated both with epilepsy and with use of antiepileptic medications. Heart. 2015;101(1):17–22. 10.1136/heartjnl-2014-305664 25031263

[9] Janszky I , Hallqvist J , Tomson T , Ahlbom A , Mukamal KJ , Ahnve S . Increased risk and worse prognosis of myocardial infarction in patients with prior hospitalization for epilepsy–the Stockholm heart epidemiology program. Brain. 2009;132(Pt 10):2798–2804. 19717532 10.1093/brain/awp216

[10] Chen Z , Liew D , Kwan P . Excess mortality and hospitalized morbidity in newly treated epilepsy patients. Neurology. 2016;87(7):718–725. 27421539 10.1212/WNL.0000000000002984 PMC4999164

[11] Mayer J , Fawzy AM , Bisson A , Pasi M , Bodin A , Vigny P , et al. Epilepsy and the risk of adverse cardiovascular events: a nationwide cohort study. Eur J Neurol. 2024;31(3):e16116. 38165065 10.1111/ene.16116 PMC11235735

[12] Wang J , Huang P , Yu Q , Lu J , Liu P , Yang Y , et al. Epilepsy and long‐term risk of arrhythmias. Eur Heart J. 2023;44(35):3374–3382. 37602368 10.1093/eurheartj/ehad523 PMC10499547

[13] Doege C , Luedde M , Kostev K . Epilepsy is associated with an increased incidence of heart failure diagnoses. Epilepsy Behav. 2021;125:108393. 34731722 10.1016/j.yebeh.2021.108393

[14] Liang D , Gardella E , Kragholm K , Polcwiartek C , Sessa M . The relationship between valproate and lamotrigine/levetiracetam use and prognosis in patients with epilepsy and heart failure: a Danish register‐based study. J Card Fail. 2022;28(4):630–638. 34438055 10.1016/j.cardfail.2021.07.020

[15] Mbizvo GK , Derry C , Davenport R . Ictal asystole – a letter within a letter. J R Coll Physicians Edinb. 2020;50(2):207–214. 10.4997/JRCPE.2020.230 32568301

[16] Mbizvo GK , Derry C , Davenport R . Ictal asystole: a diagnostic and management conundrum. J R Coll Physicians Edinb. 2019;49(2):128–131. 31188342 10.4997/JRCPE.2019.209

[17] van der Lende M , Surges R , Sander JW , Thijs RD . Cardiac arrhythmias during or after epileptic seizures. J Neurol Neurosurg Psychiatry. 2016;87(1):69–74. 10.1136/jnnp-2015-310559 26038597 PMC4717443

[18] Verrier RL , Schachter SC . The epileptic heart syndrome: epidemiology, pathophysiology and clinical detection. Epilepsy Behav Rep. 2024;27:100696. 39184194 10.1016/j.ebr.2024.100696 PMC11342885

[19] Seyal M , Pascual F , Lee CY , Li CS , Bateman LM . Seizure‐related cardiac repolarization abnormalities are associated with ictal hypoxemia. Epilepsia. 2011;52(11):2105–2111. 10.1111/j.1528-1167.2011.03262.x 21906052 PMC3203996

[20] Park KJ , Sharma G , Kennedy JD , Seyal M . Potentially high‐risk cardiac arrhythmias with focal to bilateral tonic‐clonic seizures and generalized tonic‐clonic seizures are associated with the duration of periictal hypoxemia. Epilepsia. 2017;58(12):2164–2171. 10.1111/epi.13934 29105057

[21] Nass RD , Motloch LJ , Paar V , Lichtenauer M , Baumann J , Zur B , et al. Blood markers of cardiac stress after generalized convulsive seizures. Epilepsia. 2019;60(2):201–210. 10.1111/epi.14637 30645779

[22] Lip GY , Brodie MJ . Sudden death in epilepsy: an avoidable outcome? J R Soc Med. 1992;85(10):609–611. 1433037 PMC1293687

[23] Hamed SA . Antiepileptic drugs influences on body weight in people with epilepsy. Expert Rev Clin Pharmacol. 2015;8(1):103–114. 25487080 10.1586/17512433.2015.991716

[24] Josephson CB , Wiebe S , Delgado‐Garcia G , Gonzalez‐Izquierdo A , Denaxas S , Sajobi TT , et al. Association of enzyme‐inducing antiseizure drug use with long‐term cardiovascular disease. JAMA Neurol. 2021;78(11):1367–1374.34605857 10.1001/jamaneurol.2021.3424

[25] Mintzer S , Yi M , Hegarty S , Maio V , Keith S . Hyperlipidemia in patients newly treated with anticonvulsants: a population study. Epilepsia. 2020;61(2):259–266. 10.1111/epi.16420 31912492

[26] Harnod T , Chen HJ , Li TC , Sung FC , Kao CH . A high risk of hyperlipidemia in epilepsy patients: a nationwide population‐based cohort study. Ann Epidemiol. 2014;24(12):910–914. 10.1016/j.annepidem.2014.09.008 25444891

[27] Zaccara G , Lattanzi S , Brigo F . Cardiac adverse effects of antiseizure medications. Expert Opin Drug Saf. 2022;21(5):641–652. 34942077 10.1080/14740338.2022.2023128

[28] Devinsky O . Effects of seizures on autonomic and cardiovascular function. Epilepsy Curr. 2004;4(2):43–46. 15562299 10.1111/j.1535-7597.2004.42001.x PMC531654

[29] Senapati SG , Bhanushali AK , Lahori S , Naagendran MS , Sriram S , Ganguly A , et al. Mapping of neuro‐cardiac electrophysiology: interlinking epilepsy and arrhythmia. J Cardiovasc Dev Dis. 2023;10(10):433. 37887880 10.3390/jcdd10100433 PMC10607576

[30] Srinivas S , Vignesh Rk B , Ayinapudi VN , Govindarajan A , Sundaram SS , Priyathersini N . Neurological consequences of cardiac arrhythmias: relationship between stroke, cognitive decline, and heart rhythm disorders. Cureus. 2024;16(3):e57159. 38681361 10.7759/cureus.57159 PMC11056008

[31] de la Torre JC . Cardiovascular risk factors promote brain hypoperfusion leading to cognitive decline and dementia. Cardiovasc Psychiatry Neurol. 2012;2012:367516. 23243502 10.1155/2012/367516 PMC3518077

[32] Al E , Stephani T , Engelhardt M , Haegens S , Villringer A , Nikulin VV . Cardiac activity impacts cortical motor excitability. PLoS Biol. 2023;21(11):e3002393. 38015826 10.1371/journal.pbio.3002393 PMC10684011

[33] Su JH , Luo MY , Liang N , Gong SX , Chen W , Huang WQ , et al. Interleukin‐6: a novel target for cardio‐cerebrovascular diseases. Front Pharmacol. 2021;12:745061. 10.3389/fphar.2021.745061 34504432 PMC8421530

[34] Zhang H , Dhalla NS . The role of pro‐inflammatory cytokines in the pathogenesis of cardiovascular disease. Int J Mol Sci. 2024;25(2):1082. 38256155 10.3390/ijms25021082 PMC10817020

[35] Li G , Bauer S , Nowak M , Norwood B , Tackenberg B , Rosenow F , et al. Cytokines and epilepsy. Seizure. 2011;20(3):249–256. 21216630 10.1016/j.seizure.2010.12.005

[36] Nardone R , Brigo F , Trinka E . Acute symptomatic seizures caused by electrolyte disturbances. J Clin Neurol. 2016;12(1):21–33. 26754778 10.3988/jcn.2016.12.1.21 PMC4712283

[37] Natelson BH , Suarez RV , Terrence CF , Turizo R . Patients with epilepsy who die suddenly have cardiac disease. Arch Neurol. 1998;55(6):857–860. 10.1001/archneur.55.6.857 9626779

[38] Falconer B , Rajs J . Post‐mortem findings of cardiac lesions in epileptics: a preliminary report. Forensic Sci. 1976;8(1):63–71. 824190 10.1016/0300-9432(76)90048-0

[39] Fialho GL , Wolf P , Walz R , Lin K . Increased cardiac stiffness is associated with autonomic dysfunction in patients with temporal lobe epilepsy. Epilepsia. 2018;59(6):e85–e90. 29697139 10.1111/epi.14084

[40] Liu Z , Thergarajan P , Antonic‐Baker A , Chen Z , Sparks PB , Lannin NA , et al. Cardiac structural and functional abnormalities in epilepsy: a systematic review and meta‐analysis. Epilepsia Open. 2023;8(1):46–59. 10.1002/epi4.12692 36648338 PMC9977759

[41] Vogels RL , van der Flier WM , van Harten B , Gouw AA , Scheltens P , Schroeder‐Tanka JM , et al. Brain magnetic resonance imaging abnormalities in patients with heart failure. Eur J Heart Fail. 2007;9(10):1003–1009. 10.1016/j.ejheart.2007.07.006 17719270

[42] Markousis‐Mavrogenis G , Noutsias M , Rigopoulos AG , Giannakopoulou A , Gatzonis S , Pons RM , et al. The emerging role of combined brain/heart magnetic resonance imaging for the evaluation of brain/heart interaction in heart failure. J Clin Med. 2022;11(14):4009. 35887772 10.3390/jcm11144009 PMC9322381

[43] Rugg‐Gunn FJ , Holdright D . Epilepsy and the heart. Br J Cardiol. 2010;17:223–229.

[44] Strzelczyk A , Adjei P , Scott CA , Bauer S , Rosenow F , Walker MC , et al. Postictal increase in T‐wave alternans after generalized tonic‐clonic seizures. Epilepsia. 2011;52(11):2112–2117. 10.1111/j.1528-1167.2011.03266.x 21933179

[45] Pang TD , Nearing BD , Krishnamurthy KB , Olin B , Schachter SC , Verrier RL . Cardiac electrical instability in newly diagnosed/chronic epilepsy tracked by Holter and ECG patch. Neurology. 2019;93(10):450–458. 31477610 10.1212/WNL.0000000000008077

[46] Cacciotti A , Pappalettera C , Miraglia F , Valeriani L , Judica E , Rossini PM , et al. Complexity analysis from EEG data in congestive heart failure: a study via approximate entropy. Acta Physiol (Oxf). 2023;238(2):e13979. 37070962 10.1111/apha.13979

[47] Hamed SA . Atherosclerosis in epilepsy: its causes and implications. Epilepsy Behav. 2014;41:290–296. 25164495 10.1016/j.yebeh.2014.07.003

[48] Li YX , Guo W , Chen RX , Lv XR , Li Y . The relationships between obesity and epilepsy: a systematic review with meta‐analysis. PLoS One. 2024;19(8):e0306175. 39121110 10.1371/journal.pone.0306175 PMC11315312

[49] Centers for Disease C, Prevention . Comorbidity in adults with epilepsy–United States, 2010. MMWR Morb Mortal Wkly Rep. 2013;62(43):849–853. 24172878 PMC4585598

[50] Ha FJ , Chong T , Cook MJ , Paratz ED . Epilepsy and cardiac arrhythmias: a state‐of‐the‐art review. JACC Clin Electrophysiol. 2024;11:217–229. 39570264 10.1016/j.jacep.2024.09.034

[51] Thijs RD , Surges R , O'Brien TJ , Sander JW . Epilepsy in adults. Lancet. 2019;393(10172):689–701. 30686584 10.1016/S0140-6736(18)32596-0

[52] Rosenow F , Winter Y , Leunikava I , Brunnert M , Joeres L , Sutphin J , et al. Relative importance of clinical outcomes and safety risks of antiseizure medication monotherapy for patients and physicians: discrete choice experiment eliciting preferences in real‐world study “VOTE”. Epilepsia. 2022;63(2):451–462. 34921391 10.1111/epi.17137

[53] Vouliotis AI , Roberts PR , Dilaveris P , Gatzoulis K , Yue A , Tsioufis K . Leadless pacemakers: current achievements and future perspectives. Eur Cardiol. 2023;18:e49. 37655133 10.15420/ecr.2022.32 PMC10466270

[54] Kirkfeldt RE , Johansen JB , Nohr EA , Jørgensen OD , Nielsen JC . Complications after cardiac implantable electronic device implantations: an analysis of a complete, nationwide cohort in Denmark. Eur Heart J. 2014;35(18):1186–1194. 10.1093/eurheartj/eht511 24347317 PMC4012708

